# Role of mass drug administration in elimination of *Plasmodium falciparum* malaria: a consensus modelling study

**DOI:** 10.1016/S2214-109X(17)30220-6

**Published:** 2017-05-26

**Authors:** Oliver J Brady, Hannah C Slater, Peter Pemberton-Ross, Edward Wenger, Richard J Maude, Azra C Ghani, Melissa A Penny, Jaline Gerardin, Lisa J White, Nakul Chitnis, Ricardo Aguas, Simon I Hay, David L Smith, Erin M Stuckey, Emelda A Okiro, Thomas A Smith, Lucy C Okell

**Affiliations:** a Centre for the Mathematical Modelling of Infectious Diseases, Department of Infectious Disease Epidemiology, and Malaria Modelling Consortium, London School of Hygiene & Tropical Medicine, London, UK; b MRC Centre for Outbreak Analysis and Modelling, Department of Infectious Disease Epidemiology, Imperial College, London, UK; c Swiss Tropical and Public Health Institute, Basel, Switzerland; d University of Basel, Basel, Switzerland; e Institute for Disease Modeling, Bellevue, WA, USA; f Mahidol Oxford Tropical Medicine Research Unit, Faculty of Tropical Medicine, Mahidol University, Bangkok, Thailand; g Centre for Tropical Medicine and Global Health, University of Oxford, Oxford, UK; h Oxford Big Data Institute, Li Ka Shing Centre for Health Information and Discovery, University of Oxford, Oxford, UK; i Harvard TH Chan School of Public Health, Harvard University, Boston, MA, USA; j Malaria Modelling Consortium, University of Washington, Seattle, WA, USA; k Institute for Health Metrics and Evaluation, University of Washington, Seattle, WA, USA; l Malaria Modelling Consortium, Bill & Melinda Gates Foundation, Seattle, WA, USA; m Kemri Wellcome Trust Research Programme, Nairobi, Kenya

## Abstract

**Background:**

Mass drug administration for elimination of *Plasmodium falciparum* malaria is recommended by WHO in some settings. We used consensus modelling to understand how to optimise the effects of mass drug administration in areas with low malaria transmission.

**Methods:**

We collaborated with researchers doing field trials to establish a standard intervention scenario and standard transmission setting, and we input these parameters into four previously published models. We then varied the number of rounds of mass drug administration, coverage, duration, timing, importation of infection, and pre-administration transmission levels. The outcome of interest was the percentage reduction in annual mean prevalence of *P falciparum* parasite rate as measured by PCR in the third year after the final round of mass drug administration.

**Findings:**

The models predicted differing magnitude of the effects of mass drug administration, but consensus answers were reached for several factors. Mass drug administration was predicted to reduce transmission over a longer timescale than accounted for by the prophylactic effect alone. Percentage reduction in transmission was predicted to be higher and last longer at lower baseline transmission levels. Reduction in transmission resulting from mass drug administration was predicted to be temporary, and in the absence of scale-up of other interventions, such as vector control, transmission would return to pre-administration levels. The proportion of the population treated in a year was a key determinant of simulated effectiveness, irrespective of whether people are treated through high coverage in a single round or new individuals are reached by implementation of several rounds. Mass drug administration was predicted to be more effective if continued over 2 years rather than 1 year, and if done at the time of year when transmission is lowest.

**Interpretation:**

Mass drug administration has the potential to reduce transmission for a limited time, but is not an effective replacement for existing vector control. Unless elimination is achieved, mass drug administration has to be repeated regularly for sustained effect.

**Funding:**

Bill & Melinda Gates Foundation.

## Introduction

Despite the gains made towards elimination of *Plasmodium falciparum* malaria in the past 15 years, many countries still have endemic transmission^1^ and are increasingly looking to new strategies to accelerate progress. Mass drug administration (MDA) involves the time-limited distribution of drugs to a target population, irrespective of infection status. It has been used only sporadically against malaria in most settings, and cluster-randomised trials, the most robust studies of the effect of MDA on transmission, are few.^2^, ^3^ However, MDA has received renewed interest as a strategy to clear chronic asymptomatic infections (NCT01872702) and rapidly reduce transmission.^4^, ^5^

In September, 2015, WHO's Malaria Policy Advisory Committee recommended for the first time the use of MDA in specific circumstances: when transmission is close to being interrupted, vector control, effective surveillance, and access to case management are at high coverage, and importation of infection is minimal; as a component of accelerated elimination in areas of the Greater Mekong Subregion, which are under threat of multidrug resistance; or for malaria epidemics or during complex emergencies.^6^ National malaria control programmes and partners need to decide what role, if any, MDA should have in control and elimination strategies. To answer this question, the best operational strategies for MDA and how best to combine MDA with other interventions need to be established.

To help with the Malaria Policy Advisory Committee's decision making, an evidence review group was established to synthesise available evidence for the effect of MDA on transmission of malaria.^7^ This synthesis included a few prospective field trials, retrospective analyses of previous MDAs, and a mathematical-model comparison analysis. Mathematical models are a useful way of assessing the knowledge accumulated from field trials of MDA and predicting how effectiveness might vary in settings where MDA has not yet been tested. The Malaria Modelling Consortium was tasked to compare findings from four established models (OpenMalaria,^8^ EMOD Disease Transmission Kernel [DTK],^9^ Imperial,^10^, ^11^ and Mahidol Oxford Tropical Medicine Research Unit (MORU]^12^, ^13^) on the effectiveness of MDA in different settings. Model development involves deciding on model structure, parameters, and assumptions, validation against epidemiological data, and assessment of uncertainties at each stage of this process.

**Research in context**

**Evidence before this study**

We did not do a search of published work, because three comprehensive reviews have been published in the past 4 years. A Cochrane review done in 2013 found two cluster-randomised trials, eight non-randomised controlled studies, and 22 uncontrolled before–after studies of mass drug administration. Most studies showed a substantial initial effect on parasitaemia. However, there was little evidence for effects beyond 6 months. Two further comprehensive reviews published in 2015 had wider inclusion criteria, and included published and unpublished work and studies not yet published. These reviews showed that mass drug administration was implicated in local elimination of malaria in some settings, particularly remote areas with small populations and low initial malaria transmission, but not in areas of higher transmission. Mass drug administration has been simulated in several mathematical modelling studies but, as in field studies, many factors—such as the number of doses given, number of rounds of treatment, choice of drugs, local malaria transmission intensity, and outcomes of interest—have varied greatly, all of which are likely to affect outcomes.

**Added value of this study**

In this study, a consortium of modelling groups investigated the degree of consensus of four established malaria transmission models in terms of the main determinants of the effect of mass drug administration and how large an effect on prevalence is likely to be achieved. In consultation with partners who are doing field trials of mass drug administration, we chose various programme options (eg, number of rounds, choice of drug) that were considered logistically feasible. We standardised many inputs and outputs of the models, such as initial slide prevalence, outcomes of interest, and implementation options. Our analysis showed—despite many differences in assumptions between the four models, for example about the underlying transmission dynamics of malaria—broad consensus between the models on how mass drug administration should be implemented to optimise effects, and the settings in which such programmes will be most effective.

**Implications of all the available evidence**

High coverage in the target population and mass drug administration in more than 1 year are important to maximise reductions in transmission. Reductions in transmission last longest in low-transmission settings but, unless elimination and prevention of reintroduction are achieved, malaria transmission will return to pre-intervention levels after the programme of mass drug administration finishes.

We report the results of the Malaria Modelling Consortium's MDA model comparison and their implications for the utility of MDA. Our aim for this analysis is to help with decisions on the relative prioritisation of MDA within wider malaria-control strategies.

## Methods

Malaria-control programmes implementing MDA need to decide on several operational factors, including the number of rounds, timing, and frequency of treatment. Furthermore, the effect of MDA can be influenced by epidemiological factors in each setting, such as malaria transmission level and infection importation rates. The Malaria Modelling Consortium collaborated with partners doing field trials of MDA to identify probable scenarios for MDA deployment and common operational choices that would need to be made within realistic logistic constraints.

First, a standard MDA intervention scenario was defined to use as a basis for comparison (table 1). This scenario consisted of two rounds of treatment per year, 5 weeks apart, with a standard regimen of dihydroartemisinin–piperaquine at 70% effective coverage, for 2 years. A standard setting was chosen (table 1), specifying 5% slide *P falciparum* parasite rate in 2–10-year-olds (*Pf*PR_2–10_) before MDA, intermediate seasonal variation in transmission with one rainy season, and no importation of infection. In further simulations, we varied operational factors that are of primary interest because they can be adjusted in an MDA programme: the number of rounds per year, the effective coverage of each round, the interval between rounds, and the duration of the MDA programme. Each model was used to do a multivariate analysis that simulated the baseline conditions with every combination of the selected parameters (table 1), producing 48 different MDA programmes. We also tested how the effect of MDA could vary depending on the local setting with respect to seasonal timing, vector control, importation of infection, and drug resistance; these analyses were done in selected models according to which were most appropriate for each setting.

**Table 1:** Model input parameters for programme options and local settings for mass drug administration

	**Standard scenario value**	**Values when varied**
**Programmatic considerations**		
Rounds of mass drug administration per year	2	3
Effective coverage* (%)	70%	30%, 50%, 90%
Coverage correlation between rounds	1† or 0‡	0 or 1
Interval between rounds	5 weeks	4 weeks, 6 weeks
Duration of programme	2 years	1 year
Time of year when mass drug administration begins	Optimum (as defined by each group) in a Zambia-like seasonality	Each month of the year
Other interventions	Insecticide-treated bednets at 80% effective coverage and access to passive treatment with artemisinin-based combination therapy at 60% throughout the simulation	Removal of vector control, simulated by a ten-fold increase in the emergence rate of adult mosquitos starting at the beginning of the year in which mass drug administrated is implemented
Choice of drug	Long-lasting artemisinin-based combination therapy with properties similar to dihydroartemisinin–piperaquine	..
**Transmission setting characteristics**		
Baseline transmission intensity	5% *Pf*PR_2–10_, as measured by microscopy	1 to 10
Importation of malaria cases	None	0·4–1·6 infections per 10 000 people per year^14^
Population size	10 000	1000
Artemisinin resistance	0%	Variable
Seasonality profile	Zambia-based single annual rainy season profile	Two rainy seasons per year, no seasonal variation in transmission

The standard intervention scenario was used as a basis for comparison and values were varied as shown. *Pf*PR_2–10_=*Plasmodium falciparum* parasite rate in children aged 2–10 years.

* Defined as the percentage of the population that takes the full course of drug that clears all parasites (the product of access to intervention, adherence, and drug efficacy). The denominator corresponds to the entire population; ineligible people (eg, pregnant women) and infants younger than 6 months are not included in mass drug administrations.

† The same people are treated in each round in the EMOD Disease Transmission Kernel, Imperial, and OpenMalaria models.

‡ Random individuals are treated in each round in the Mahidol Oxford Tropical Medicine Research Unit model.

The outcome metric was the percentage reduction in annual mean all-age prevalence of *P falciparum* as measured by PCR (*Pf*PR_PCR_) in the third year after the final round of MDA. The models assessed represent four different ways of simulating malaria transmission and MDA, and a summary of their characteristics and functionality is given in table 2.^11^, ^14^, ^15^, ^16^, ^17^, ^18^, ^19^, ^20^, ^21^, ^22^, ^23^, ^24^, ^25^, ^26^, ^27^, ^28^, ^29^

**Table 2:** Summary of models of malaria transmission

	**EMOD DTK**	**Imperial**	**MORU**	**OpenMalaria**
Institutional home	Institute for Disease Modelling	Imperial College London	Mahidol Oxford Tropical Medicine Research Unit	Swiss Tropical and Public Health Institute
Type of model and references	Individual-based stochastic microsimulation^16^, ^17^	Individual-based stochastic microsimulations of malaria in human beings linked to a stochastic compartmental model for mosquitoes^11^	Deterministic compartmental model described by differential equations,^18^ including drug action on each stage of the infection	Single-location individual-based simulation of malaria in human beings^14^ linked to deterministic model of malaria in mosquitoes^19^
How infections are tracked	Tracks parasite densities of different surface-antigen types	Tracks membership of categories of infection (symptomatic, asymptomatic, submicroscopic, treated)	Tracks membership of categories of infection	Tracks parasite densities corresponding to different infection events
Relationship between entomological innoculation rate and prevalence	Immunity is acquired through cumulative exposure to different antigenic determinants,^20^ with heterogeneity in individual biting rates included	Immunity is acquired through cumulative exposure to mosquito bites, with heterogeneity in individual biting rates included	Subdivides population into non-immune and immune classes	Submodels of infection of human beings^14^ and of blood-stage parasite densities, with main immune effects controlling parasite densities^21^
Duration of infections	Infection duration based on malaria therapy^20^ and cross-sectional survey data^22^	Infection duration based on fitting to asexual parasite prevalence data by age, transmission intensity, seasonality	Infection duration based on malaria therapy data and data from endemic areas	Infection duration based on malaria therapy data^21^
Effect of mass drug administration or case management	Reduces blood-stage parasite densities according to age-specific and dose-specific pharmacokinetics and pharmacodynamics,^22^ with corresponding clearance and prophylactic effects	Truncates infections and has subsequent prophylactic effect based on fitting pharmacokinetic and pharmacodynamic models to field studies	Post-treatment prophylactic period derived from field studies of time to next infection	Truncates infections, and has subsequent prophylactic effect based on pharmacokinetic and pharmacodynamic studies
Validation against trials of mass drug administration or mass screening and treatment	Assessed against MACEPA trial of mass screening and treatment in southern Zambia^23^	Assessed against a controlled trial^24^ of mass drug administration in Burkina Faso (model slightly optimistic about effect *vs* data), and the MACEPA trial of mass screening and treatment in southern Zambia (model matched data)	Fitted to a trial of mass drug administration in Cambodia^25^	Fitted to the data of the Garki project (Matsari),^26^ and assessed against the MACEPA trial of mass screening and treatment in southern Zambia^27^
Infectiousness to mosquitoes	A function of mature gametocyte and cytokine densities^20^, ^22^	Related to asexual parasite dynamics and lagged to allow for development of gametocytes	Infected individuals have a constant infectiousness	Lagged function of asexual parasite density^28^
Heterogeneity in exposure	Age-dependent biting^29^ and configurable distribution of household variability (the latter disabled in this analysis)	Included	Not included	Included
Initial state	..	Back-calculating required mosquito density to achieve given initial prevalence at an approximate steady state in the presence of treatment and long-lasting insecticide-treated nets	Set transmission rate to achieve given initial prevalence at an approximate steady state in the presence of treatment	Back-calculating required mosquito density to achieve given initial prevalence at an approximate steady state in the presence of treatment
Source of seasonality pattern	Rainfall and imputed temperature^29^ driving larval habitat model fitted to clinical incidence patterns in Sinazongwe and Gwembe districts, Zambia	Rainfall data from Zambia combined with larval and adult mosquito model	Same entomological innoculation rate input as Imperial model	Based on pattern for southern Zambia^29^
Age-structured model	Yes	Yes	No	Yes
Simulation of correlated rounds of intervention	Yes	Yes	No	Yes

All the models are extensible to include other functionality (eg, different drugs, effects of drug resistance, effect on drug resistance, vector bionomics and details of vector control, different initial conditions, other concomitant interventions). A detailed comparison of EMOD DTK, Imperial, and OpenMalaria, including references to the data to which they are fitted, is available elsewhere.^15^ DTK=Disease Transmission Kernel. MORU=Mahidol Oxford Tropical Medicine Research Unit. MACEPA=Malaria Control and Elimination Partnership in Africa.

### Role of the funding source

EMS and EAO are or have been employed by the study funder, and were involved in data collection, analysis, and interpretation, and writing of the Article. However, they were not involved in study design The funder had no further role in study design; data collection, analysis, or interpretation; or writing of the Article. The corresponding author had access to all study data and was responsible for the final decision to submit for publication.

## Results

**Figure 1:**
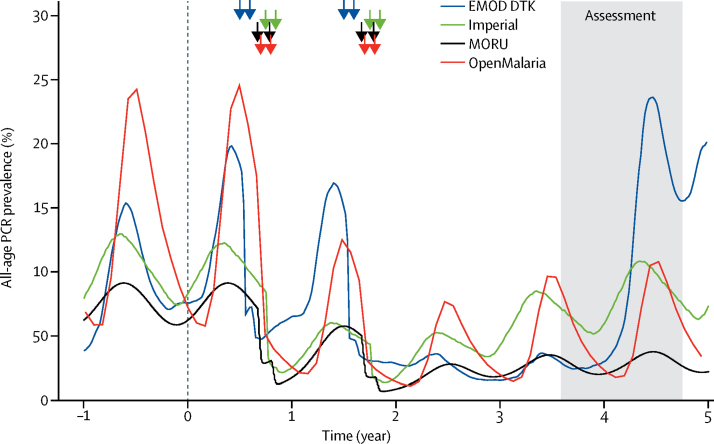
Sample simulated output from four different models of effect of mass drug administration on all-age PCR prevalence of *Plasmodium falciparum* infection From year −1 to year 0, the models are at equilibrium. The timing of each round of mass drug administration in each model is shown by coloured arrows. The four different models show the output under the standard intervention scenario (70% coverage, 2 years of mass drug administration, two rounds per year, 5 weeks between rounds, seasonal transmission [based on Zambia], and 5% mean annual prevalence pre-intervention by microscopy in 2–10-year-olds). DTK=Disease Transmission Kernel. MORU=Mahidol Oxford Tropical Medicine Research Unit.

In all four models, simulated prevalence fell substantially immediately after MDA (figure 1) as a result of successful clearance of infection and the prophylactic effect of piperaquine, a long-acting artemisinin-combination therapy partner drug. However, in the absence of elimination, the models predicted that prevalence of infection would thereafter return to pre-intervention levels (at different rates in different models). After the prophylactic effect of the partner drug has declined, the key factors that determine local transmission intensity, such as the density of mosquitoes, have not been permanently changed. Thus, without some other long-term intervention, such as improved vector control, the effects of MDA were predicted to be transient.

**Figure 2:**
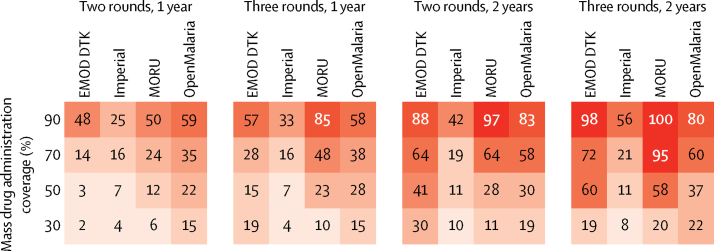
Percentage reduction in mean annual all-age PCR prevalence of *Plasmodium falciparum* in the third year after mass drug administration Numbers in boxes are percentage reductions. The darker the colour, the larger the reduction. DTK=Disease Transmission Kernel. MORU=Mahidol Oxford Tropical Medicine Research Unit.

Although the four different models showed similar trends in the effects of MDA with time, substantial differences were noted in both simulated pre-intervention transmission and the magnitude of the effect (Figure 1, Figure 2). Pre-intervention *Pf*PR_PCR_ differed between models because we standardised these initial conditions to 5% slide prevalence in 2–10-year-olds, and the models make several different assumptions, for example about the relation between slide and PCR prevalence. The EMOD DTK and MORU models predicted a reduction in *Pf*PR_PCR_ of 64% in the third year after MDA, OpenMalaria a reduction of 58%, and Imperial a reduction of 19%. These differences were caused by many different assumptions, including the relation between entomological inoculation rate, prevalence, and the basic case reproduction number (R_0_); the effect of case management; the degree of stochastic variability; and the dynamics of immunity.

Despite the differences in effect size predicted by the different models, there was generally greater agreement as to the relative effect of different operational choices. Effective coverage had a large effect on percentage reduction in *Pf*PR_PCR_ in all models. For example, the median percentage reduction in *Pf*PR_PCR_ in the standard scenario at 30% coverage was 15% (range across models 10–30); at 70% coverage, it was 61% (range 19–64; figure 2). Duration of intervention was also important in all models, with prevalence estimated to remain low for longer with 2 years of MDA than with 1 year of MDA (figure 2). When multiple rounds of MDA are done, all models showed that coverage overlap substantially affects MDA. For example, if participation was entirely random in each round, two rounds of MDA at 70% coverage would reach roughly 90% of the population with one or more treatment courses. However, if the same individuals participated in each round, then two rounds at 70% coverage would reach only 70% of the population. In reality, the situation is likely to be somewhere between the two extremes, and strategies that specifically target individuals missed in the first round are likely to be more effective as long as these strategies do not come at the expense of maximising the total number of individuals treated per year.

**Figure 3:**
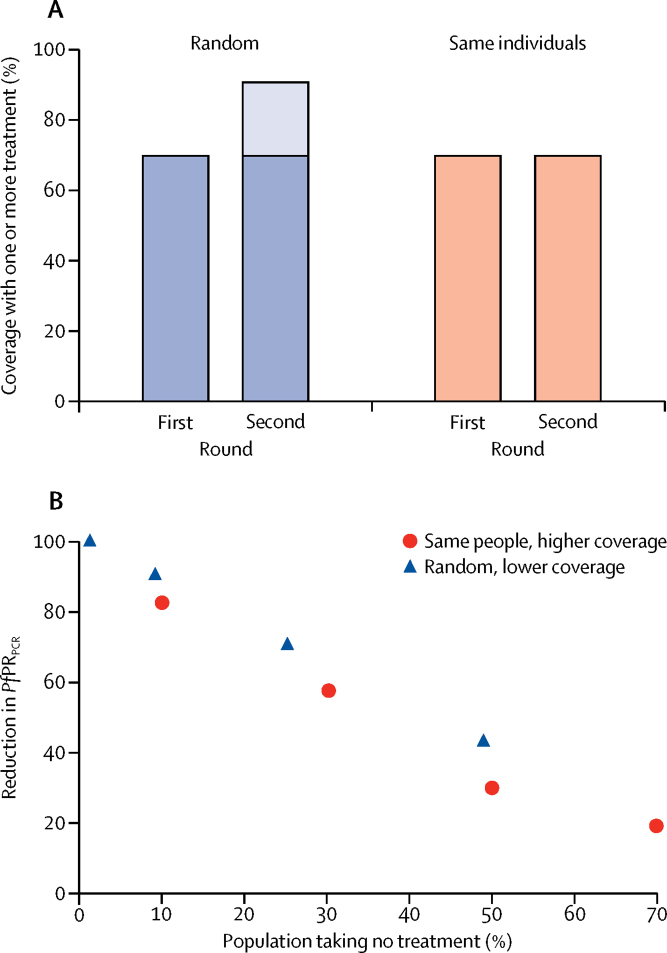
Overlap in coverage between rounds of mass drug administration and effect on *Pf*PR_PCR_ (A) shows the proportion of the population receiving one or more treatment courses after two rounds of mass drug administration, each at 70% coverage, with either random participation or the same individuals participating each time. (B) shows the percentage reduction in *Pf*PR_PCR_ 3 years after mass drug administration according to the proportion of the population not receiving treatment in any rounds in the baseline scenario. Blue triangles represent two rounds of mass drug administration randomly targeted at 30%, 50%, 70%, and 90% coverage; red dots represent the same two rounds of mass drug administration in which the same individuals are treated in each round. Results shown are from the OpenMalaria model. *Pf*PR_PCR_=*Plasmodium falciparum* parasite rate as measured by PCR.

All four models suggested that, with closely spaced rounds of MDA (ie, intervals of 4–6 weeks), the most important operational factor determining effect is the proportion of the population who do not receive any treatment in any round (figure 3). Implementation of three rather than two rounds of treatment per year had negligible effects if the same individuals participated in each round at intervals of 4–6 weeks, but resulted in better outcomes if additional people were reached in the third round who had not previously received treatment that year. Spacing within-year MDA rounds 4 weeks apart rather than 6 weeks apart made little difference in terms of overall effects.

In settings with one rainy season a year, all models predicted greater reductions in *Pf*PR_PCR_ if MDA was done during the dry season or at the end of the rainy season, as previously found in similar analyses by the separate models.^12^, ^13^, ^30^, ^31^, ^32^ In the Imperial model, reductions in *Pf*PR_PCR_ were as much as 1·45 times greater if MDA was done in the dry season or at the end of the rainy season than at the beginning of the rainy season. Seasonal timing has less of an effect on *Pf*PR_PCR_ in the OpenMalaria and MORU models. In a setting with two rainy seasons per year, we predicted that timing of MDA had less of an effect, because transmission was more evenly spread throughout the year. At a given mean baseline slide prevalence, MDA was predicted to be marginally more effective in a seasonal setting (at the optimum time) than in a non-seasonal setting.

The introduction of MDA while vector control was simultaneously removed led to a sudden and large increase in simulated all-age prevalence, and the subsequent MDA programme did very little to reduce this shift even in the short term (OpenMalaria and Imperial models). We predicted, therefore, that an MDA programme of this type is insufficient to replace vector control, even at the highest levels of coverage.^33^

In a high prevalence setting (ie, 10% *Pf*PR_2–10_), the percentage reduction in *Pf*PR_PCR_ was considerably lower in all the models compared with that in a setting with 5% prevalence. We predicted that, even with high coverage (90%), three rounds per year, and 2 years of intervention, *Pf*PR_PCR_ 3 years later would be reduced by only a median of 48% (range across models 19–95) from its pre-intervention level, compared with 80% (range across models 56–100) in the setting with 5% baseline prevalence. This disparity is because transmission rebounds more rapidly in a higher transmission area. However, the absolute reduction in transmission is greater in higher prevalence settings because more infections are cleared.

**Figure 4:**
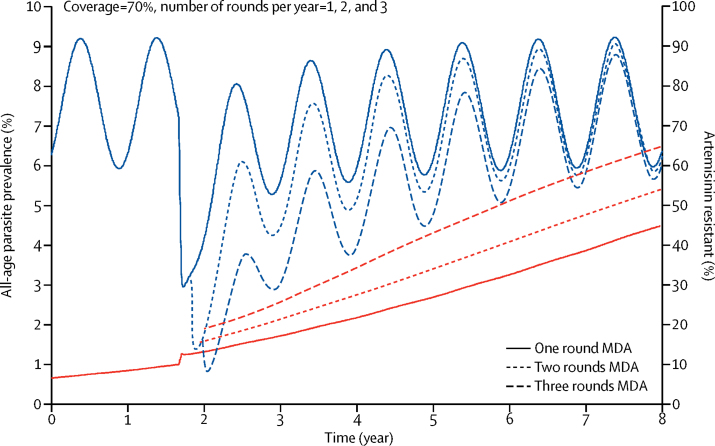
Effect of MDA with artemisinin-combination therapy on malaria prevalence and the percentage of parasites that are artemisinin resistant Results shown are from the MORU model. Blue lines show parasite prevalence, whereas red lines show the percentage artemisinin resistant. Coverage was 70% per round. MDA=mass drug administration. MORU=Mahidol Oxford Tropical Medicine Research Unit.

When PfPR_2–10_ is 5%, cases imported at a low rate (as in the WHO-recommended MDA use scenarios), based on data from Zanzibar,^34^ represent a very small proportion of the total infections in the population, and therefore make little difference to MDA effectiveness. However, when PfPR_2–10_ is lower, imported cases would be instrumental to increasing transmission. MDA more easily caused stochastic extinction in smaller than in bigger simulated populations in all the models. Finally, some evidence (only tested with the MORU model) suggested that MDA with artemisinin-combination therapies could speed up the selection of artemisinin-resistant parasite strains (figure 4). However, the size of this effect could be limited by the high selection pressure already imposed from management of symptomatic cases.

## Discussion

Although individual models predicted different magnitudes of the effects of MDA, we found substantial consensus on which factors have the greatest influence on these effects, including both the characteristics of the programme and the setting in which MDA is applied. Percentage reductions were predicted to be highest in low-transmission settings and smaller populations, but were more transient in other settings, in line with evidence from field studies.^2^, ^3^ Infection importation rates (when transmission is not very low) and the spacing between rounds (within the 4–6 week range examined) had little effect. The proportion of the population reached by at least one round of MDA per year and the duration of the programme had a large influence on effectiveness, and our analysis suggested that these factors should be the focus of operational efforts.

We did not formally analyse which differences between the models created the variation in predicted effects of MDA. MDA can substantially affect transmission in the short term, leading to different transmission dynamics from those analysed in in-depth comparisons of models of the effects of RTS,S.^15^ Differences between the models in basic epidemiological quantities, including duration of untreated infections and clinical immunity, could be relevant. Different levels of within-population heterogeneity in malaria exposure are assumed, which are crucial for the stability of transmission in low-prevalence settings. Seasonal variation makes transmission less stable at a given prevalence, whereas spatial heterogeneity can make transmission more stable. We did not include spatial heterogeneity in transmission levels, but it is likely to be crucial at very low transmission levels. Simulations of small subpopulations with 5–10% prevalence, diluted by other subpopulations of unexposed individuals, might be appropriate representations of large populations with 1% average prevalence. The size of such subpopulations and their degree of interconnection could then be crucial, because stochastic extinction is much more likely in smaller than in larger populations. Consideration of spatial structure in the models will be crucial for more realistic modelling of malaria elimination. In each model, the initial prevalence for simulations was fixed, but this value could correspond to very different epidemiological patterns, for example, of the immune status of human beings or vectorial capacity.

A key next step for the modelling groups is to continue using data from forthcoming trials of MDA to further validate the models and to continue efforts to understand how and why model predictions differ, such as under the HIV, tuberculosis, and neglected tropical diseases model consortiums.^35^, ^36^ Use of modelling to understand the potential role of MDA in containing outbreaks after elimination, and to compare the predicted effects of MDA on drug resistance, will also be important.

The value of our simulations is that they show that, despite many differences in assumptions, there is a consensus between models on the relative influence of MDA operational characteristics. Many of our results accord with findings from MDA for lymphatic filariasis,^37^, ^38^, ^39^ although caution should be taken in extrapolating findings between the diseases in view of the generally much higher reproductive number and shorter generation time of malaria. Our results form one part of a broad evidence base, including growing evidence from malaria field trials, which should be considered when policy makers decide whether MDA is a useful strategy for their settings. This reassessment should balance the predicted benefits of MDA against equivalent investments in existing interventions, while considering other consequences such as the risk of spreading resistance. Under no circumstances did any of the models predict that MDA is an effective replacement for vector control, and indeed the overarching message from this model comparison is that, without some other sustained change, such as improved vector control, the effects of MDA are likely to be transient. When MDA is implemented, sustainability of the programme and maintenance of other interventions will be major challenges to ensure long-term reduction in malaria burden.
